# Procedure for the Selection of Rubber Compound in Rubber-Metal Springs for Vibration Isolation

**DOI:** 10.3390/polym12081737

**Published:** 2020-08-03

**Authors:** Milan Banić, Dušan Stamenković, Aleksandar Miltenović, Dragan Jovanović, Milan Tica

**Affiliations:** 1 Faculty of Mechanical Engineering, University of Niš, 18000 Niš, Serbia; dusan.stamenkovic@masfak.ni.ac.rs (D.S.); aleksandar.miltenovic@masfak.ni.ac.rs (A.M.); dragan.s.jovanovic@masfak.ni.ac.rs (D.J.); 2 Faculty of Mechanical Engineering, University of Banja Luka, 78000 Banja Luka, Bosnia and Herzegovina; milan.tica@mf.unibl.org

**Keywords:** rubber-metal spring, selection of rubber compound, virtual experiment, numerical simulation, vibration isolation

## Abstract

The selection of a rubber compound has a determining influence on the final characteristics of rubber-metal springs. Therefore, the correct selection of a rubber compound is a key factor for development of rubber-metal vibration isolation springs with required characteristics. The procedure for the selection of the rubber compound for vibration isolation of rubber-metal springs has been proposed, so that the rubber-metal elements have the necessary characteristics, especially in terms of deflection. The procedure is based on numerical simulation of spring deflection with Bergström-Boyce constitutive model in virtual experiment, with a goal to determine which parameters of the constitutive model will lead to spring required deflection. The procedure was verified by case study defined to select rubber compound for a rubber–metal spring used in railway engineering.

## 1. Introduction

Rubber or rubber-metal springs have a wide industrial application as elements for damping shock and vibration loads and load distribution between the supports. The rubber-metal spring assembly consists of at least two metal plates/cylinders bonded with each other with natural or synthetic rubber using a vulcanization process or injection molding. Noted design allows to take advantage of both assembly components: high ability to deform and absorb energy by rubber and high surface load resistance by metal parts.

Until the advent of modern software tools, the development of rubber-metal products (and other products made of elastomers) was based on the previous experience of the designer and the method of “trial and error”. Such an approach is inefficient, expensive, and time consuming as it requires an iterative procedure with numerous experimental checks to achieve the required product characteristics.

The emergence of the finite element method and the development of models for predicting the mechanical behavior of rubber compounds (constitutive models) have significantly improved the process of development of rubber-metal springs. The mechanical behavior of elastomers is described through hyperelastic models of almost incompressible materials in which the ratio of stress and strain in the material is determined through the derivative of the deformation energy density function.

Integration of hyperelastic models into commercial FEM (Finite Element Method) software enables stress and strain verification (even static stiffness) [1,2,3,4], fatigue life [5,6,7], and fracture (crack) [5,8], as well as optimization geometry of elements from the aspect of increasing life expectancy [6,9].

It was not possible to estimate hysteresis damping, thermo-mechanical effects, relaxation phenomena, Payne (Fletcher-Gent), and Mullins effect by using hyperelastic models. With the advent of rubber viscoelastic/viscoplastic constitutive models, it was possible to overcome the limitations of hyperelastic models and new fields of application of the finite element method in the development of rubber-metal springs have been opened. The implementation of these models in software packages has enabled engineers to evaluate dynamic stiffness [10], spring hysteresis [11,12,13], internal heat generation [14,15,16,17], and related elastomer aging [14,18].

However, in most cases, the nonlinear dynamic behavior of rubber-made elements is neglected or at best significantly simplified [19]. Only a few authors used a transient modal decomposition analysis [20], generalized Maxwell model [21], to predict transmissibility and dynamic response. Despite the evident progress in available engineering tools, the methodology of development of rubber and rubber-metal springs is still based on physical prototyping and testing [22], i.e., on previous experience of the designer and trial and error methods. Most of the authors dealt with individual aspects of the mechanical behavior of the rubber without considering how to combine numerous new procedures into a unified development methodology. The selection of rubber compound for the spring is based on general recommendations about applicability of base rubber for specific application [23], Shore hardness [24] and extensive experimental testing to achieve required stiffness properties. The authors use simulations only to determine mechanical response, optimize the geometry with a goal to increase the life of the component and predict heat generation. But all noted research is performed with a known rubber compound for which the constitutive model parameters are determined experimentally.

This paper presents a novel procedure for selection of rubber compound for rubber–metal spring based on parametrization of rubber constitutive model constants in virtual experiment performed by finite element method. The virtual experiment goal is to find required spring deflection at certain strain speed and load for all possible rubbers existing in the previously formed database of rubber compounds and their constitutive model parameters. The procedure uses a Bergström–Boyce constitutive model, as a viscoelastic material model, to predict the mechanical behavior of the rubber-metal spring due to its prediction accuracy and ability to capture dependence of mechanical response from strain speed. The procedure provides fast and reliable selection of rubber compound and it is verified by preforming the procedure for a rubber–metal element used in railway engineering.

## 2. Theory and Procedure Description

The novel procedure for selection of rubber compound for rubber–metal spring was defined to enable systematic approach to compound selection, by employing numerical simulation, in order to obtain required mechanical response, with a goal to reduce the physical prototyping and testing.

### 2.1. Vibration Isolation with Rubber-Metal Spring

Rubber-metal springs belong to the group of passive vibration isolators. Unlike steel springs, which can be represented by a simplified oscillator without damping, rubber-metal springs have hysteresis (structural) damping. The natural frequency (*f_n_*) of a system with one degree of freedom of movement in rubber-metal springs is a function of the ratio of the mass of the system (*m*) and the dynamic stiffness of the spring (*k_dyn_*), as presented in Equation (1):

(1) $$f_{n}=\frac{1}{2\pi }\sqrt{\frac{k_{dyn}}{m}}$$

The performance of vibration isolators can be assessed through transmissibility (*T*), which is defined as the ratio of inputs and outputs (energy, force, displacement, and acceleration). Transmissibility is defined as a function of the excitation frequency (*f*), natural frequency (*f_n_*), and the damping factor (*ζ*), as presented in Equation (2):

(2) $$T=\sqrt{\frac{1+4\zeta^{2}r^{2}}{{\left(1-r^{2}\right)}^{2}+4\zeta^{2}r^{2}}}$$

where *r* is a tuning factor defined as ratio of excitation and natural frequency, Equation (3):

(3) $$r=\frac{f}{f_{n}}$$

As the damping factor for rubber-metal springs is relatively small (*ζ* < 0.1), the existence of damping is often neglected in practice and the calculation is performed under the assumption that the damping factor is *ζ* = 0.

The selection of rubber-metal spring comes down to the selection of the desired transmissibility and damping, i.e., only transmissibility if the damping is neglected. Then, it is possible to determine the system natural frequency for the known excitation frequency and the predetermined tuning factor based on expression Equation (2). The tuning factor should be larger than √2 to ensure that vibration isolation instead of amplification is achieved.

As the spring natural frequency is now known, from the condition that dynamic stiffness is 1.1–1.4 larger than static stiffness (*k*) [1] and the generally known relation on the value of spring stiffness, Equation (4):

(4) $$k=\frac{m\cdot g}{s}$$

it is possible to determine the required static spring deflection (*s*) by transforming Equation (4) into Equation (5) [25]:

(5) $$s=\frac{\left(1.1-1.4\right)\cdot 0.248}{{\left(f_{n}\right)}^{2}}$$

Equation (5) shows that for desired transmissibility it is only necessary to determine the spring static deflection. This is widely used in practice to define rubber for vibration isolators as the prototypes of springs are made with different rubber compounds to achieve required static spring deflection with experimental testing.

### 2.2. Bergström–Boyce Constitutive Model

One of the most used viscoelastic constitutive models used in predicting the mechanical behavior of elastomers is the Bergström-Boyce material model. To overcome the previously described problems of hyperelastic models with the accuracy of predicting the mechanical behavior of elastomers, as well as the linearity of classical viscoelastic models, Bergström and Boyce proposed a modification of Zener’s material model. Bergström-Boyce is a phenomenologically based nonlinear model of a material that can predict the viscoelastic behavior of elastomers. The model can predict the nonlinear dependence between stress and strain, the dependence of the mechanical response on the strain rate and the occurrence of hysteresis in elastomers. According to Bergström [26], the time-dependent mechanical behavior of real elastomers can be described by splitting into two parts: parallel networks: the equilibrium response (network A) and the time-dependent deviation from the equilibrium response (network B).

The model gives equations for calculation the total Cauchy stress of the networks A and B by using the eight-chain Arruda-Boyce constitutive model [26]. The constitutive model is defined by nine constants defined in Table 1 which are determined by curve fitting procedure with elastomer experimental data.

The limiting stretch of network A and B are usually considered equal, so the model is then reduced to eight material constants. Bergström himself [27] showed that his model has sufficient precision to predict the mechanical behavior of elastomers at least up to a filler concentration of 25 vol.% in natural and chloroprene rubbers. Bergström [28] further directly compared the Yeoh and Arruda-Boyce models with the Bergström-Boyce model. In this study, the author shows that the Bergström-Boyce model achieves significantly higher accuracy in predicting the mechanical response of elastomers compared to Yeoh and Arruda-Boyce models, even if they are extended with the Ogden-Roxborough or Ki-Boyce damage model. It has also been shown that the prediction of the mechanical response of the Bergström-Boyce model extended with the Ogden-Roxborough (Bergström-Boyce-Mullins) or Ki-Boyce damage model almost coincides with the experimentally determined mechanical response (difference below 2% in relation to the coefficient of determination *R*^2^). Other authors [29,30,31] also proved the accuracy of prediction of Bergström-Boyce model. The model is very accurate up to strains larger than 0.3 and there is a slight deviation of the predicted behavior from experimentally determined above the noted strain value.

### 2.3. Novel Procedure for Selection of Rubber in Rubber-Metal Springs

**Figure 1:**
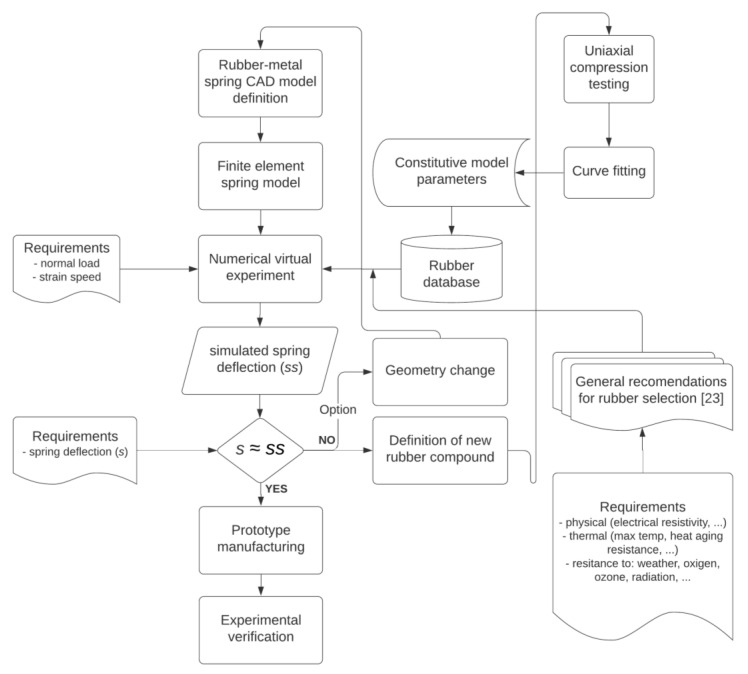
Procedure for selection of rubber compound in rubber-metal vibration isolators.

Figure 1 shows the novel procedure of selecting a rubber compound for rubber-metal vibration isolators, based on the idea of applying approach that would provide an answer to the question: which parameters of the Bergström-Boyce material model (or any other viscoelastic constitutive model) will result with required mechanical response, i.e., deflection of the rubber-metal spring at defined strain speed and load?

Each rubber compound has a unique set of constitutive model parameters, so by testing of every rubber compound available, it is possible to form a database of compounds with constitutive model parameters. Then, it is possible to define a virtual experiment to determine spring deflection at certain load and strain speed for all possible rubbers satisfying requirements regarding physical and thermal properties, as well as the resistance to various conditions. Noted additional requirements are checked based on general recommendations defined in [23] and only appropriate rubber compounds are selected for the virtual experiment.

When the appropriate set of constitutive parameters is found for which the spring have a required deflection at given load and strain speed, the prototype of the rubber-metal spring can be manufactured and tested.

If no rubber with a unique set of constitutive parameters has a required static deflection, there is a possibility to define a new rubber compound as shown in Figure 1, by analysis of the results of the virtual experiment. The rubber candidate with deflection closest to required deflection could be made stiffer or more elastic by increase/decrease of filler content. As the filler content is known for all compounds, as well as their deflection based on virtual experiment, a simple interpolation can be used to determine which filler content would result with required static deflection. Another option would be to parametrize the geometry of the spring and to modify the spring deflection by changing of geometry parameters for the rubber compound with the closest deflection required.

One of the basic research ideas is to replace complicated standard testing procedures (uniaxial, biaxial, and plane stretching, as well as stress relaxation testing), which are most often performed in specialized accredited laboratories, by simple uniaxial compression on a universal testing machine. Compression testing is far simpler to perform than standard tests and can be performed in any laboratory that has a universal testing machine. Uniaxial compression is a simple alternative to biaxial stretching because no special sample testing tool is required. Also, unlike tensile testing, an extensometer is not required when compressing a specimen to determine deformation of the specimen.

The described procedure can be applied using any viscoelastic/viscoplastic constitutive elastomer model with sufficient mechanical behavior prediction accuracy, if the data on the parameters of that model are available in the rubber database. Furthermore, the procedure could introduce numerous goals apart equality of static deflection, such as achieving of required hysteresis and damping, transmissibility, heat generation, etc. In principle, all important rubber-metal spring parameters can be used to find rubber compound constitutive parameters with virtual experiment for a set of requirements.

To fully explain the above-mentioned compound selection procedure, as well to perform its verification, the procedure was carried out for the SERBIA Cargo series 441/444 locomotive central bolt rubber-metal spring. The noted spring is manufactured by numerous companies such as Trelleborg, Continental, etc. and its geometry is well known and defined by locomotive manufacturer ASEA from Sweden (now ABB). The locomotive manufacturer defined that the spring should have a required deflection in axial and radial direction as given in Table 2, which summarizes the requirements for series 441/444 locomotive central bolt rubber-metal spring. The requirement regarding the compression speed given in Table 2 was defined according to EN 13913:2008 [32] which defines testing of rubber-metal springs used in railway applications.

## 3. Materials and Experiments

### 3.1. Testing of Rubber Compounds

As already explained above, the necessary precondition for the application of a systematized process of rubber compound selection is the formation of a database on rubber compound. The database of compounds from which it is possible to make rubber-metal springs is formed based on data obtained by mechanical testing of rubber compounds.

The rubber compounds were made by request from research team by the company “TIGAR technical rubber” (Pirot, Serbia). The basic criterion when choosing compounds was to use rubbers that are commonly used for the production of rubber-metal springs, so that elastomers from the group of natural rubber, isobutene-isoprene rubber (butyl rubber) and acrylonitrile-butadiene rubber of different hardness were selected. Basic composition data supplied from manufacturer “TIGAR technical rubber” for all compounds used in research are given in Table 3. All compounds used in the research are designated by their trade name which represents the internal designation of the manufacturer. In addition to the compounds that “TIGAR technical rubber” already uses in manufacturing of rubber-metal springs, a requirement is specified to make two more compounds with a natural rubber base whose hardness should be around 60 and 70 IRHD by modification of compound with trade name A-615 (hardness 65 IRHD) by decreasing (A-615’–60 IRHD) or increasing (A-615″–70 IRHD) the carbon black content. Mechanical characteristics of the selected compounds are given in Appendix A and they are supplied by the manufacturer also. As compounds A-615’ and A-615″ do not exist in the standard production program of the company, only the hardness value is delivered for them.

The testing program of rubber mixtures is defined based on general recommendations for compression testing.

All experimental tests were performed on a universal test machine Shimadzu AGS-10kNXD (Kyoto, Japan) between hardened steel plates. The specification of the experimental test is given in Table 4, which defines the number of specimens per compound and its size, environmental conditions, and universal testing machine settings. The effect of friction on the resulting force and change in the shape of the sample was reduced by achieving tribological conditions that allowed the base of the sample to slide with minimal resistance on compressing steel plates.

**Figure 2:**
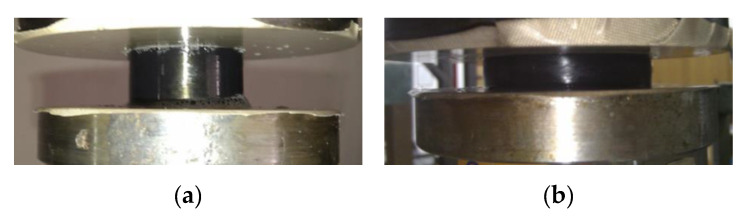
Rubber sample in: (**a**) unloaded and (**b**) compressed state.

Prior to testing, samples of the compound were conditioned at ambient temperature for at least 24 h. The samples were placed between compression plates coated with Teflon foil, as shown in Figure 2a. A mild aqueous soap solution was applied to both the sample and the compression plates. By applying Teflon coating and lubricating the coated plates and the sample, a minimal impact of friction on the test process is achieved. The sample retains its shape in the compressed state, as shown in Figure 2b.

Each sample was conditioned by compression 5 times to a maximum deformation of 9 mm, at a compression speed of 0.1 mm/s without recording a mechanical response. At maximum deformation, the sample was held for 60 s, after which it was unloaded. Upon completion of conditioning, the samples were stabilized for 30 min. After the rest period, all samples were tested according to the procedure described in Table 5, which defines testing procedure for capturing of mechanical response of compounds necessary for extraction of constitutive model parameters. During the test, data on the value of force and deflection were acquired to obtain the mechanical response of the sample.

### 3.2. Numerical Simulations

The numerical model was defined in accordance with the test conditions for the central bolt rubber-metal spring given in Table 2. 3D CAD model of the central bolt was made based on available technical documentation and reverse engineering using 3D scanning. The CAD model of the rubber-metal spring is transferred from the CAD software to the ANSYS software (Canonsburg, PA, USA) for finite element method simulations. Two static structural analysis were defined which correspond to axial and radial compression of the central bolt. As there is a symmetry of geometry and load in both axial and radial direction, only a quarter model was used to define axial load case and one half of the model was used for a radial load case.

The material data for cylindrical steel parts was taken from ANSYS material database for structural steel. The constants of the rubber compound material model (Bergström-Boyce) were defined as discrete variables and a table of parameter values was formed for each of the rubber compound from the compound database which is applicable according to requirements. As all compounds in the database satisfy the applicability criterion, they were all transferred to ANSYS software. The Bergström-Boyce material model is accessible in ANSYS only via the command interface, where variable material parameters were defined as arguments as shown in Appendix A-Figure A1. In this way, a plan of a virtual experiment was defined.

Since the aim of the analysis is to determine the spring deflection, the discretization of the model was performed by applying higher order finite element SOLID186 [33] with a coarse mesh. Such approach greatly increases the analysis speed, as it was proven that deformation quickly converges to final simulation value with coarse meshes [25]. If the goal of the analysis is to find required transmissibility or damping value, the higher quality mash should be used. The full integration method was used to solve the stiffness matrix.

**Figure 3:**
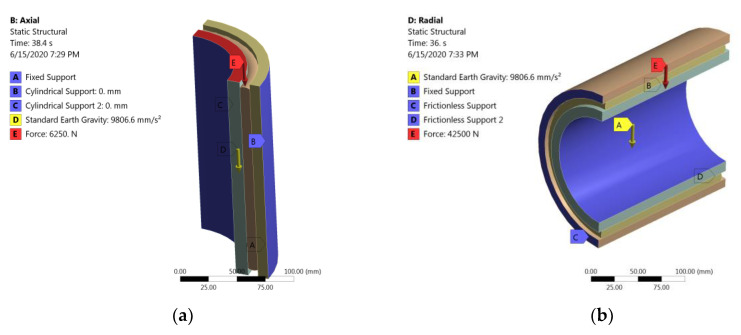
Finite element model and load and boundary conditions for axial (**a**) and radial (**b**) load cases.

Figure 3 shows the finite element model with loads and boundary conditions for axial and radial load case.

Within postprocessing, the deflection of the spring in the axial and radial directions is considered as the output parameter of the virtual experiment.

## 4. Results

### 4.1. Results of Testing of Rubber Compounds

**Figure 4:**
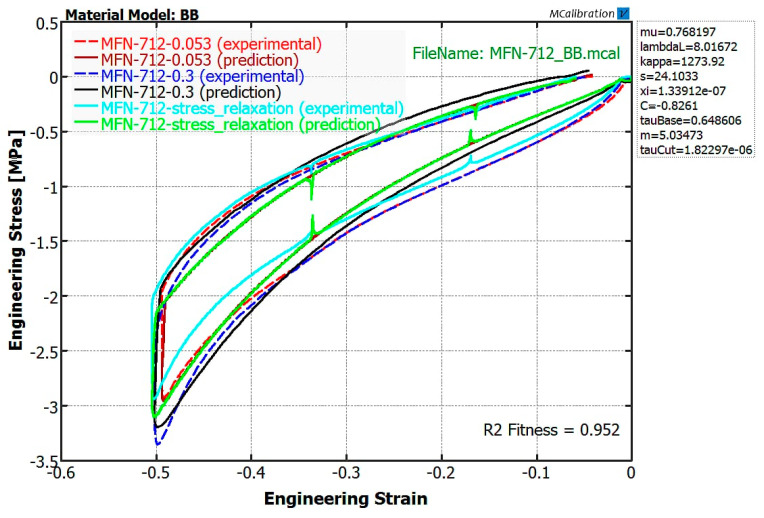
Result of curve fitting for the B-712 compound with Bergström-Boyce constitutive model.

Based on the obtained force–deflection diagrams, with the assumption of incompressibility of the samples, i.e., constant volume during the test, the samples engineering stress were calculated. The experimentally determined mechanical behavior for all rubber compounds was transferred to the MCalibration software (Needham, MA, USA) where the load cases were adjusted according to the conditions corresponding to the experimental test (temperature, conditioning, and load direction). Based on the experimental data, the parameters of the Bergström-Boyce constitutive model were determined in the MCalibration by curve fitting procedure. Model parameters were determined by maximizing the coefficient of determination *R^2^* value between the model predictions and experimental data. One of the curve fitting results is shown on Figure 4, where comparison between experimental data and model predictions is given for B-712 compound.

The determined constitutive model constants by curve fitting procedure given in Table 6 are shown according to extended ANSYS Bergström-Boyce model notation [33] and they correspond to command interface implementation of Bergström-Boyce constitutive model shown in Appendix A-Figure A1. It was considered that $\lambda_{A}^{lock}=\lambda_{Be}^{lock}$, so the model is reduced to eight constitutive model constants.

The determined constitutive model parameters shown in Table 6 were stored in the rubber compound database for use in later stage of compound selection procedure, i.e., numerical simulations.

### 4.2. Results of Numerical Simulations

The results of virtual experiment performed by numerical simulation for axial and radial load case are shown in Table 7. The spring deflection was determined in radial and axial direction for all the rubber compounds in the database.

## 5. Discussion and Procedure Verification

From the analysis of results given in Table 7, one can conclude that for the specific shape of the rubber-metal spring used in procedure verification (locomotive central bolt) the axial deflection is much more dependent upon the compound type than the radial one. This can be explained by the fact that in the axial direction the rubber has enough space to change it shape. In the radial direction the rubber is confined between metal cylinders, so, in this case, geometry has more influence on deflection than the compound type.

Based on the results of the virtual experiment given in Table 7, one can determine that there are two rubber compounds which satisfy the requirements given in Table 2. Compounds A–615 and A–615′ have a slightly larger deflection in axial direction than required (3.2 mm), but within allowed tolerance of 10%. As for radial deflection, both compounds have deflection which is very close to required one. Between two candidate compounds, the decision was made to manufacture the spring prototype from A–615 compound, as it is a known compound with proven record of performance and resistance to damaging effects in railway environment.

It is possible that none of the compounds used in virtual experiment would result with deflection in axial and radial direction which are within acceptable tolerances. As it was already noted in procedure description, there is a possibility to define new rubber compound (Figure 1) by analysis of the results of the virtual experiment. For instance, if we neglect the candidate compounds, the rubber compounds with deflection closest to required deflection are A–515 and A–615″. They are on the opposite side of the required spring deflection—compound A–515 is too elastic and the compound A–615″ is too stiff. Those are compounds with same base elastomer (natural rubber) and with different fraction of carbon black filler which defines their stiffness. In the same group of base elastomers, as the quantity of filler content is known to compound manufacturer, he could determine from virtual experiment the dependence of deflection from the quantity of filler. Then it is possible to define new rubber compound within the same group of base elastomers with appropriate filler content which would result in required spring deflection.

If it is observed that, like in this specific case, the deflection is more dependent to geometry than rubber compound selection, it is better to optimize the geometry of the spring by defining the virtual experiment with parametrized dimensions of the spring for a known rubber compound.

**Figure 5:**
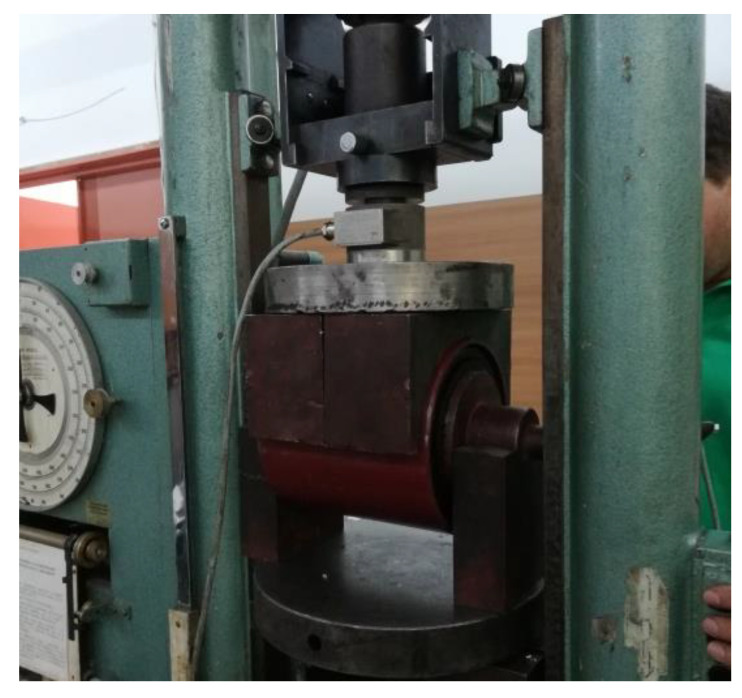
Experimental testing of radial deflection for a central bolt spring manufactured from A–615 compound.

Several spring prototypes were manufactured from compound A–615 and tested on a uniaxial testing machine to verify the procedure for selection of rubber compound. Figure 5 shows the testing of manufactured rubber-metal spring in radial direction where the spring is positioned in a tool designed for radial testing. The testing was performed according to test specification given in Table 2.

The results of manufactured prototypes testing are given in Table 8. One can conclude that the manufactured rubber-metal spring deflection is within the requested specification. There is a slight deviance between the results of the virtual experiment for the compound A–615 and the experimental results. The deviance is within 10% which is a very good agreement between numerical simulations and experimental testing in elastomer simulations. It is interesting that axial deflection was predicted larger than the experimental one, while the radial deflection was underestimated.

The validation results show that it is possible to select rubber compound by usage of virtual experiment with a defined analysis goal (in this case required spring deflection), where constitutive model parameters are used as experimental variables. The presented procedure is fast and computationally efficient, as the results are obtained within several hours even with large number of rubber compounds existing in the compound database. Although, formation of compound database requires significant effort to perform testing of multiple compounds and determine constitutive model parameters, the usability of the procedure in practical engineering justifies the made efforts. Another strength of the proposed procedure is the possibility to employ any viscoelastic/viscoplastic model available which would even increase the procedure’s accuracy in compound selection if more advanced material models are employed (such as Bergström-Boyc-Mullins, Three Network Model, etc.). However, the noted models require implementation of user material (UMAT) in finite element software and there is a performance penalty associated with their use. For compounds used for the manufacturing of rubber-metal springs, the Bergström-Boyce material model has a satisfactory precision in predicting of mechanical response, thus it is sufficient for the presented procedure.

## 6. Conclusions

This paper defines a systematic procedure for selecting a rubber compound based on the required deflection of the spring, i.e., natural spring frequency, unlike the classic process of development of rubber-metal springs in which the selection of a rubber compound was made primarily on the basis of the previous experience of the designer.

The presented procedure assumes that it is possible to select a rubber compound by definition of constitutive model parameters as discrete input variables in an FE virtual experiment targeted towards obtaining the required spring deflection. The procedure is based on application of the Bergström-Boyce constitutive model whose accuracy of prediction of mechanical response is very high for compounds used for the manufacturing of rubber-metal springs. The constitutive model parameters for the virtual experiment are taken from the compound database, which is formed by extracting of constitutive model parameters by a curve fitting procedure from mechanical response of a simple cylindrical specimen. Mechanical response is captured by uniaxial compression which is far simpler to perform than standard tension tests necessary to capture the viscoelastic mechanical response.

The procedure was verified by case study defined to select rubber compound for a rubber-metal spring of locomotive central bolt. Nine different rubber compounds provided by the manufacturer of rubber-metal springs were tested and a preliminary compound database with Bergström-Boyce constitutive model parameters was formed. By application of the procedure presented in the paper, the compound for manufacturing of the spring prototype was selected. The spring prototype was manufactured and tested, and it was determined that the prototype has the required axial and radial deflection as predicted by results of the numerical virtual experiment for a selected compound.

The presented procedure is a first step towards unified methodology for development of rubber-metal springs. Further research should be directed towards increasing the number of rubber compounds in the database, application of advanced material models with a damage model, and validating the procedure when the compound selection is based on other requirements such as transmissibility, damping, heat generation, permanent set, etc. Such an approach would lead to complete prediction of spring performance in operating conditions before the prototype is actually made, which is of extreme importance for practical engineering.

## Figures and Tables

**Table 1:** Material constants of the Bergström–Boyce constitutive model.

Designation	Material Model Parameter
$\mu_{A}^{0}$	initial shear modulus of the network A
$\lambda_{A}^{lock}$	limiting stretch of the network A
$\mu_{B}^{0}$	initial shear modulus of the network B
$\lambda_{Be}^{lock}$	limiting stretch of the network B
ξ	strain adjustment factor
${\hat{\tau }}_{B}$	flow resistance
*m* $\in R^{+}$	stress exponential
C∈[0,−1]	strain exponential
*K*	bulk modulus

**Table 2:** Requirements for the series 441/444 locomotive central bolt rubber-metal spring.

Element	Pieces per Locomotive	Load Direction	Load, kN	Compression Speed, mm/min	Required Deflection (*s*), mm
Rubber element of the central bolt	2	axial	25	5	3.2 mm ± 10%
		radial	85	1	0.6 mm ± 10%

**Table 3:** Composition of compounds manufactured by company “TIGAR technical rubber”.

Compound. Trade Name	Rubber Matrix	Carbon Black Particle Grade, ASTM	Carbon Black Content, phr	Other Ingredients for All Compounds
AC–502/4	nitrile rubber	N375	20	Zinc Oxide Sulphur Stearin Antioxidant Sulfonamide Tiuram
A–515	natural rubber	N375	10	
AC–609/3	nitrile rubber	N375	20	
A–615	natural rubber	N375, N550	25, 10	
A–615′	natural rubber	N375, N550	20, 10	
A–615″	natural rubber	N375	30	
B–712	butyl rubber	N375	24	
A–715	natural rubber	N375, N550	30, 15	
A–815	natural rubber	N375, N550	45, 25	

**Table 4:** Specification of experimental uniaxial compression of rubber samples.

Test Condition	Value
Dimensions of a rubber sample	Ø35.7 ± 0.5 × 17.8 ± 0.5 mm
Ambient temperature during experiments	23 ± 2 °C
Maximum compression force	10 kN (depending on the elastomer in experiment)
Maximum stoke	9 mm
Number of samples per one mixture	3
Test machine control method	automatic machine stroke control based on internal displacement sensor feedback

**Table 5:** Test procedure for rubber specimens.

Step	Procedure
1	From the initial position, the load is performed with a speed of 0.1 mm/s to the maximum deformation (9 mm) and then the sample is unloaded with the same speed until complete relief
2	From the initial position, the load is performed with a speed of 1 mm/s to the maximum deformation (9 mm) and then the sample is unloaded with the same speed until complete relief
3	From the initial position, loading is performed with a speed of 0.1 mm/s to one third of the maximum deformation (3 mm), where it is retained for 20 s; the load is continued at the same speed up to two thirds of the maximum deformation (6 mm), where it is retained for 20 s; the load is continued at the same speed until the maximum deformation (9 mm). Without holding at the same speed, the sample is unloaded in reverse order

**Table 6:** Determined constants of Bergström–Boyce constitutive model.

Compound Trade Name	ARG1 $\begin{array}{l} \mu_{A}^{0},\\ {N/m}^{2} \end{array}$	ARG2 $\begin{array}{l} {\left(\lambda_{}^{lock}\right)}^{2},\\ N^{2}{/m}^{4} \end{array}$	ARG3 $\begin{array}{l} \mu_{B}^{0},\\ {N/m}^{2} \end{array}$	ARG4 $\begin{array}{l} \frac{1}{{\hat{\tau }}_{B}^{m}},\\ m^{2}/N \end{array}$	ARG5 *C*	ARG7 *m*	ARG8 ξ	ARG8 $\frac{1}{Κ}$
AC–502/4	628,866	22.857	2,488,599	1.196 × 10^−12^	−0.880	2.207	0.074	9.573 × 10^−10^
A–515	704,728	70.305	832,071	1.307 × 10^−13^	−0.273	2.455	0.154	1.646 × 10^−9^
AC–609/3	6,020,450	69.146	3,188,899	1.661 × 10^−11^	−0.298	2.065	0.164	2.144 × 10^−9^
A–615	763,702	103.264	58,342,098	1.207 × 10^−10^	−0.059	1.799	0.426	2.823 × 10^−9^
A–615′	771,376	78.693	5,376,738	4.549 × 10^−12^	−0.268	2.113	0.138	2.369 × 10^−9^
A–615″	988,747	92.686	8,816,937	6.123 × 10^−14^	−0.233	2.405	0.164	1.281 × 10^−9^
B–712	768,197	64.268	18,516,061	5.473 × 10^−30^	−0.826	5.035	1.34 × 10^−8^	7.85 × 10^−10^
A–715	1,043,490	91.080	13,692,376	6.196 × 10^−13^	−0.259	2.119	0.191	1.331 × 10^−9^
A–815	1,630,510	88.641	193,001,838	1.392 × 10^−21^	−0.060	3.706	0.389	2.721 × 10^−9^

**Table 7:** Results of the virtual experiment performed by axial and radial load case.

**Compound Trade Name**	**Deflection in Axial Direction, mm**	**Deflection in Radial Direction, mm**
AC–502/4	4.28	0.61
A–515	3.81	0.59
AC–609/3	4.51	0.63
A–615	3.51	0.59
A–615′	3.52	0.58
A–615″	2.69	0.52
B–712	2.42	0.48
A–715	2.52	0.51
A–815	1.32	0.44

**Table 8:** Test results of rubber-metal elements for locomotive 441/444 made from compound A–615.

Rubber–Metal Element	Required Characteristics	Number of Samples	Measured Deflection at Maximum Force (mm)		Force *F*_1_/*F*_2_, kN	Stiffness, kN/mm
Rubber element of the central bolt	Axial deflection F = 25 kN *s* = 3.2 mm ± 10%	2	3.37	3.45	5–25	6.152 5.614
	Radial deflection F = 85 kN *s* = 0.6 mm ± 10%	1	0.64		20–80	30.020

**Figure A1:**
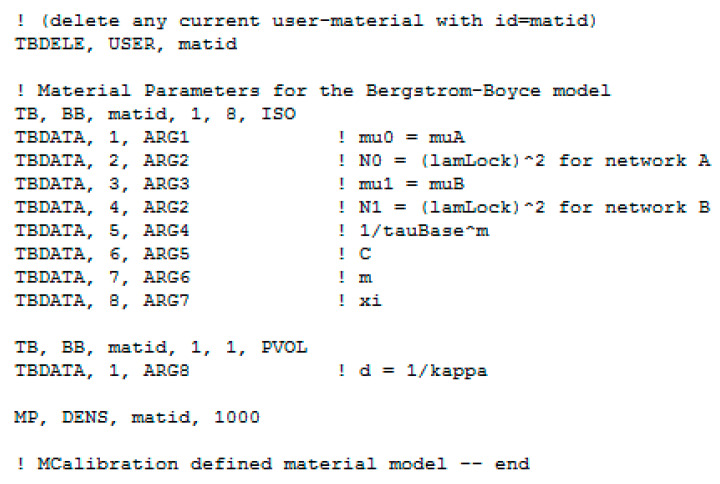
Implementation of Bergström–Boyce constitutive model via the ANSYS software command interface.

## Appendix A

**Table A1:** Mechanical properties of compounds used in research.

Property	Unit	Standard	AC-502/4	A-515	AC-609/3	A-615	A-615′	A-615″	B-712	A-715	A-815	Test Conditions
Density	g/cm^3^	–	1.033	–	1.048	–	–	–	–	–	–	no aging
Hardness	IRHD	ISO 48-2	55	57	59	65	61	69	80	72	82	no aging
			55	57	60	66	–	–	–	71	84	after aging
Modulus 200	MPa	ISO 37	2.7	5.4	4.3	9.7	–	–	9	12.5	/	no aging
			3.0	6.0	4.7	10.1	–	–	–	13.1	/	after aging
Modulus 300	MPa		4.9	10.4	7.9	17.3	–	–	–	20.4	/	no aging
			5.7	11.2	8.9	17.7	–	–	–	/	/	after aging
Tensile ultimate strength	MPa		13.7	24.0	9.5	21.7	–	–	15.3	21.9	17.3	no aging
			12.8	20.4	10	20.7	–	–	–	18.7	18.8	after aging
Stretching (when torn)	%		511	442	332	349	–	–	379	317	176	no aging
			460	405	317	334	–	–	–	268	174	after aging
Permanent set	%	ISO 815-1	5.8	9.6	5.8	9.6	–	–	12.1	9.6	9.6	no aging
			17.14	16.1	1.49	16.1	–	–	–	16	16	after aging
